# Prevalence and Correlates of Sexually Transmitted Infections in Transgender People: An Italian Multicentric Cross-Sectional Study

**DOI:** 10.3390/jcm11102774

**Published:** 2022-05-14

**Authors:** Carlotta Cocchetti, Alessia Romani, Francesca Mazzoli, Jiska Ristori, Filippo Lagi, Maria Cristina Meriggiola, Giovanna Motta, Marina Pierdominici, Alessandro Bartoloni, Linda Vignozzi, Mario Maggi, Alessandra Daphne Fisher

**Affiliations:** 1 Andrology, Women’s Endocrinology and Gender Incongruence Unit, Careggi University Hospital, Viale Pieraccini 6, 50100 Florence, Italy; carlotta.cocchetti@gmail.com (C.C.); alessiaromani@hotmail.it (A.R.); francesca.mazzoli@stud.unifi.it (F.M.); jiska.ristori@unifi.it (J.R.); linda.vignozzi@unifi.it (L.V.); 2 Infectious and Tropical Diseases Unit, Careggi University Hospital, 50134 Florence, Italy; filippo.lagi@unifi.it (F.L.); alessandro.bartoloni@unifi.it (A.B.); 3 Gynecology and Physiopathology of Human Reproduction, IRCCS Azienda Ospedaliero-Universitaria di Bologna, 40138 Bologna, Italy; cristina.meriggiola@unibo.it; 4 Department of Medical and Surgical Sciences (DIMEC), University of Bologna, 40138 Bologna, Italy; 5 Division of Endocrinology, Diabetology and Metabolism, Department of Medical Sciences, University of Turin, 10126 Turin, Italy; giovanna.motta.83@gmail.com; 6 Center for Gender-Specific Medicine, Italian National Institute of Health, 00138 Rome, Italy; marina.pierdominici@iss.it; 7 Endocrinology Unit, Department of Experimental and Clinical Biomedical Sciences “Mario Serio”, Careggi University Hospital, University of Florence, 50139 Florence, Italy; m.maggi@dfc.unifi.it

**Keywords:** transgender, sexuality, sexually transmitted infections, HIV, stigma, epidemiology

## Abstract

The burden of sexually transmitted infections (STIs) in the transgender population remains an underestimated issue. The aims of the present study were to evaluate the prevalence of either self-reported and serological STIs and to describe socio-demographic and clinical characteristics of transgender individuals with STIs. A consecutive series of 705 transgender individuals (assigned-male at birth, AMAB *n* = 377; assigned-female at birth, AFAB *n* = 328) referring to six Italian gender clinics were included. Sociodemographic and clinical information was collected during the first visit. In a subsample of 126 individuals prevalence of STIs (human immunodeficiency virus, HIV; hepatitis C, HCV; hepatitis B, HBV; syphilis) were evaluated through serology tests. The self-reported prevalence of HIV, HBV, HCV and syphilis infection in the total sample were 3.4%, 1.6%, 2.6% and 2.0%, respectively. In the subsample who underwent serological tests, higher rates of serological prevalence were found (9.5%, 4.0%, 5.6% and 7.9% for HIV, HBV, HCV and syphilis, respectively). When comparing transgender people with or without self-reported STIs, unemployment, previous incarceration, justice problems and sex work resulted more frequent in the first group (*p*< 0.03 for all). Regarding health status, we observed higher rates of lifetime substance abuse and psychiatric morbidities in trans people with at least one reported STI (*p* < 0.05). The prevalence of STIs exceeded that reported in general population and STIs correlates underline the importance of stigma and discrimination as determinants of transgender health.

## 1. Introduction

Individuals whose gender identity does not match the assigned sex at birth may describe themselves as trans or transgender (AMAB and AFAB for those assigned male or female at birth, respectively) [1]. During their life, trans people may experience stigmatization and discrimination in many contexts [2,3]. It is well known that stigma represents an important determinant of health [4], leading to increased risk of marginalization, sex work for economic survival, incarceration, drug abuse and limited health care access [5,6,7,8]. These are traditionally considered risk factors for sexually transmitted infections (STIs). In fact, STIs spread represents one of the most relevant—although underestimated [9,10]—clinical issues in trans people. To date, very few studies evaluated the prevalence of STIs in this population, focusing on HIV and sex workers’ subsample [11,12,13,14,15,16,17]. In a recent review and meta-analysis, Baral and colleagues showed a worldwide HIV prevalence of 19% in trans AMAB people, with a 49-fold increased odds of HIV infection compared with cisgender persons of reproductive age [18]. On the other hand, Van Gerwen and colleagues recently documented in a systematic review an HIV prevalence among trans AFAB people ranging from 0% to 8.3% [19]. However, little is known about the prevalence of non-HIV STIs, such as viral hepatitis and syphilis. In addition, STIs spread among trans AFAB people remains a poorly studied topic. Furthermore, the epidemiology of STIs in trans people in Italy has not yet been defined, leading to difficulty in implementing prevention strategies and promoting adequate social and medical support. Considering that STIs are associated with a severe risk of chronic complications, studying this topic is extremely important. The present study’s main objective is to evaluate the prevalence of both self-reported and/or serological STIs (HIV, viral hepatitis and syphilis) in a large sample of trans people referring to six Italian gender clinics. Furthermore, since an extensive evaluation of sociodemographic and clinical features of trans people with STIs is lacking in the current literature, we aimed to evaluate the association between STIs and these characteristics.

## 2. Materials and Methods

### 2.1. Participants

Individuals referring for the first time to several Italian gender clinics (Florence, Rome, Bologna, Turin, Cagliari and L’Aquila) between February 2007 and January 2020 were enrolled in the present cross-sectional study. The aforementioned gender clinics are centers with expertise in the assessment of gender incongruence/dysphoria, providing psychological support and hormonal/surgical affirming treatments. Transgender people were both self-referred and referred from general practitioners for psychological or medical support related to their gender incongruence.

Inclusion criteria were as follows: age older than 18 years, condition of gender dysphoria (GD) according to the DSM 5 criteria established at the time of the referral [20]; whereas exclusion criteria included linguistic barrier, intellectual disability and differences of sexual development (DSD).

A total of 24 subjects was excluded from the initial sample because of the following reasons: drop out during clinical assessment (*n* = 19) and absence of GD (*n* = 5, one had internalized homophobia, one transvestite fetishism and three personality disorder). The final selected sample (AFAB, *n* = 328; AMAB *n* = 377) included 293 participants from Florence, 208 from Rome, 146 from Bologna, 45 from Turin, 8 from L’Aquila and 5 from Cagliari. All subjects provided their written informed consent, and the study was carried out in accordance with the ethical standards of the responsible institutional committees.

### 2.2. Measures

Data were collected during the first visit through a face-to-face structured interview, part of the routine clinical assessment, by health care professionals specialized in this field, mainly psychologists. In particular, subjects were asked about several sociodemographic parameters, smoking status, lifetime medication use, substance abuse, sex work, justice problems and personal/familiar medical and psychological morbidities. Furthermore, information was also collected regarding previous gender-affirming hormonal treatment, surgical interventions and current pharmacological treatment. To explore all these features, standard questions were used, codifying the answers as a dummy variable (no/yes, 0/1).

Sociodemographic features and health status differences were analyzed between trans people who reported at least one STI and trans people who did not (*n* = 48 and *n* = 657, respectively). This was made in order to evaluate the largest sample of individuals, instead of limiting the analysis to individuals with a serological assessment.

Furthermore, a subsample enrolled after May 2007 in the Florence gender clinic underwent serological assessment for HIV, HBV, HCV and syphilis infections at the time of the referral. HIV screening was performed by ELISA and reactive samples were subsequently confirmed by Western blot. Syphilis infection (past or present) was determined by non-treponemal and treponemal assays (Veneral Disease Research Laboratory, VDRL; *Treponema pallidum* hemagglutination assay, TPHA). For epidemiological purposes, a sample was considered positive if at least one test resulted positive. HBV infection was assessed by the presence of surface antigen (HbsAg) and anti-core antibody (anti-HBc), determined using ELISA. According to current guidelines [21], HBV infection was defined by the presence of HBsAg, HBcAb or both. In contrast, HBV immunity due to vaccination was defined by the presence of HBsAb alone. To determine HCV infection, HCV antibodies IgG-IgM were tested by ELISA.

All participants were informed about the results and individuals with a positive diagnosis were referred to specialized centers to receive proper evaluation and treatment.

### 2.3. Statistical Analysis

Data are expressed as median (quartiles) when not normally distributed, as mean ± standard deviation (SD) when normally distributed and as a percentage when categorical. The Chi-square test was used for categorical variables. Post hoc paired contrasts with Tukey’s B test were performed for the pairwise comparison among the groups. Moreover, unpaired two-sided Student’s *t*-tests were used for comparison of means of normally distributed parameters. Binomial logistic regression analysis was used to investigate the association of categorical outcome variables with categorical or continuous independent variables, including age as a covariate considering that the assessed features are generally considered age-correlated. All statistical analyses were performed on SPSS 25 for Windows (SPSS Inc., Chicago, IL, USA).

## 3. Results

### 3.1. Self-Reported Prevalence of STIs

A total of 705 transgender individuals (AMAB *n* = 377 and AFAB *n* = 328) was included in our sample, with a sex ratio of 1.15:1. The mean age at the first access to our clinics was 30.81 ± 10.09 years. AMAB people were significantly older than AFAB people (mean age 32.24 ± 10.78 and 29.17 ± 8.97, respectively; *p* < 0.0001), thus all the following results were adjusted for age.

**Figure 1:**
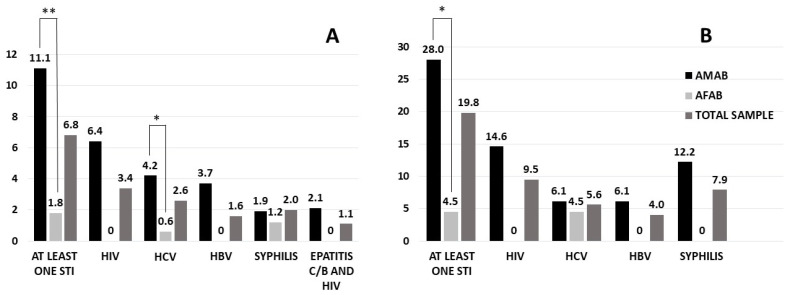
Self-reported (**A**) and serological (**B**) prevalence of STIs in transgender people. Comparison between AMAB and AFAB trans people has been reported after adjusting for age. * *p* = 0.05 and ** *p* < 0.0001. AMAB = assigned male at birth; AFAB = assigned female at birth.

Information regarding self-reported prevalence is reported in Figure 1A. The self-reported prevalence of at least one STI (among syphilis, HIV, HBV and HCV) was 6.8% in the whole sample, significantly higher in AMAB compared to AFAB people (*p* < 0.0001). Regarding isolated HCV infection, we found a prevalence of 2.6%, with higher rates in AMAB compared to AFAB people (*p* = 0.03, after adjusting for age). The 3.4% of the total sample reported HIV infection, whereas the 1.1% reported a hepatitis C/B and HIV co-infection. Furthermore, the HBV and syphilis infection prevalence in the whole sample were 1.6% and 2.0%, respectively. Interestingly, except for HCV infection, all the aforementioned differences between AMAB and AFAB individuals were lost after adjusting for age.

### 3.2. Serological Prevalence of STIs

Among the subsample in which serology tests were assessed (AFAB *n* = 44 and AMAB *n* = 82), HIV infection was found in 9.5% individuals. The prevalence of HBV, HCV and syphilis in the whole sample was 4.0%, 5.6% and 7.9%, respectively. No statistically significant differences regarding HIV, HBV, HCV and syphilis prevalence were found between AMAB and AFAB individuals after adjusting for age. Figure 1B shows the serological prevalence of each STI in this subsample. A new STI diagnosis was made only in six individuals (one for HCV, HBV and HIV, respectively, three for syphilis) who did not know/mention having a STI during the face-to-face interview.

### 3.3. Sociodemographic Characteristics

The main sociodemographic characteristics of our sample are reported in Table 1.

Since trans people with at least one self-reported STI were significantly older than trans individuals without STIs (39.42 ± 8.42 vs. 30.19 ± 9.92, respectively, *p* < 0.0001), all the following results have been adjusted for age. When evaluating sociodemographic features, no statistically significant differences were observed between the two groups with regard to marital status and education. However, more often, trans people with at least one STI were AMAB and 31.3% were not Italian natives. The majority was from South America (*n* = 11), whereas one patient was from North Africa, two from the Philippines and one from another European country. Besides, the percentage of people taking gender-affirming hormonal treatment at the time of the first referral was significantly higher in trans people with STIs. Other significant differences were found between the two groups. Mainly, unemployment was more frequent in trans people with STIs. Moreover, trans people with STIs reported higher rates of previous incarceration, justice problems and sex work. When evaluating reasons for sex work, 72.3% reported economic reasons, whereas 27.7% reported other underlying reasons (i.e., assuming a feminine gender role).

### 3.4. Lifestyle, General and Mental Health Status

Concerning lifestyle related-features and health status, no significant differences were observed between the two groups with respect to medical morbidities, smoking status, alcohol assumption and current medication use. However, trans people with STIs reported higher rates of history of substance abuse (particularly opium and cocaine) and psychiatric disorders (in particular, depression and personality disorder). A significantly higher percentage of trans individuals with STIs reported sexual abuse during adolescence, whereas no differences were observed between the two groups regarding self-harming, bullying and suicide attempts. All the aforementioned results are reported in Table 2.

**Figure 2:**
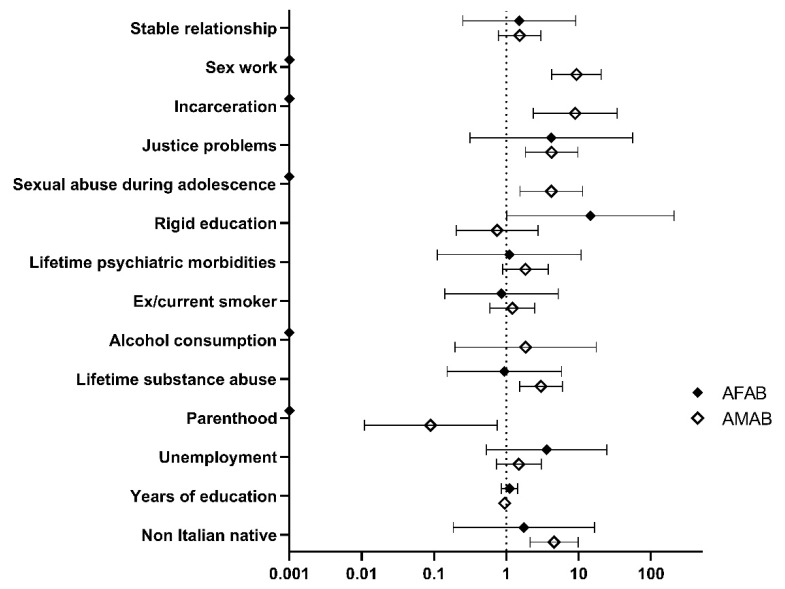
Age-adjusted odds ratios for STIs in AMAB and AFAB trans people. AMAB = assigned male at birth; AFAB = assigned female at birth.

Figure 2 summarizes the age-adjusted odds ratios (OR) for STIs separately in AMAB and AFAB trans people, as derived from the logistic regression analysis. Among evaluated sociodemographic characteristics, in trans AMAB people there was an increased OR for having at least one STIs for being not-Italian native (OR = 4.58, 95% CI 2.14–9.84), previous incarceration (OR = 8.97, 95% CI 2.36–34.11), sex work (OR = 9.31, 95% CI 4.22–20.55), justice problems (OR = 4.23, 95% CI 1.84–9.75), sexual abuse during adolescence (OR = 4.20, 95% CI 1.55–11.36) and lifetime substance abuse (OR = 3.02, 95% CI 1.53–5.95; *p* < 0.01 for all).

## 4. Discussion

To the best of our knowledge, this is the first study assessing the reported and serological STI prevalence in a large sample of Italian transgender people, focusing on the possible socio-demographic correlates in this population. Moreover, our study addressed not only HIV infection but also other STIs, such as HCV, HBV and syphilis.

The main results of the present study were the following: (i) we confirmed a higher prevalence of STIs in AMAB vs. AFAB trans people; however, this association did not retain statistical significance after adjusting for age; (ii) serology performed on a subsample of participants disclosed six new diagnosis of STI; (iii) in AMAB trans subjects, having at least one STI was associated with a foreign country of origin, unemployment, commercial sex work, history of substance abuse and justice problems.

Regarding AMAB individuals, the serological HIV, HBV and HCV infection rates (14.6%, 6.1% and 6.1%, respectively) were in line with those reported By Luzzati et al. [10] in an Italian sample of AMAB transgender individuals referring for gender affirming surgery (12.1%, 4.6% and 3.7%, respectively), while a previous meta-analysis described a HIV prevalence of 21·6% in trans AMA people in high-income countries, with an odds ratio of 48.8 in comparison with the general adult population [18]; on the other hand, Luzzati et al. reported no HIV-infected individual among trans AFAB people, and this result was confirmed by our study [10].

Even if higher STI rates in AMAB trans people are widely described [5,9,10,16,22], age has never been taken into account as an influencing factor. Indeed, the here reported relevance of age in influencing gender-prevalence differences can be related to the higher marginalization and transphobia experienced by AMAB trans people in the past years [23]. However, as the information on age at the time of STI diagnosis is not available, conclusions cannot be drawn on this regard. Moreover, we should also consider that AMAB trans people were older compared to AFAB ones and this represents per se a risk factor to encounter any STI. Furthermore, the serological prevalence of at least one STI was higher than the reported one. The latter might be a result of the lack of awareness about ways of transmission of STIs, but also of an “HIV optimism”. In fact, the success of antiretroviral medication in controlling HIV infection could induce an underestimation of the importance of safe sex, opening the way to the spread of HIV itself, as well as other STIs [24]. In this regard, in our sample, we reported six new diagnosis of STI, which is a small but relevant proportion of individuals: in fact, ignoring to be a carrier of a STI might lead to the unwanted/unintentional spread of the disease to sex partners, as well as to the development of serious complications. Similar findings were reported by Clements-Nolle and colleagues, describing many HIV-positive trans persons of their study sample not knowing to be infected and considering themselves at low-risk of contracting HIV [9]. Apart from the lack of awareness, the internalized STI-related stigma or the fear of being rejected from gender-affirming procedures may affect the STI-status disclosure. Thus, our results highlighted that the initial assessment of trans people at their first referral to gender clinics should include questions about sexual behaviors and history, in order to identify those needing a serological screening for STIs [9,10,25].

After trying to give a picture of the STIs diffusion in an Italian trans people sample, we aimed at describing the possible demographic features associated with unsafe sex and other risk behaviors among transgender individuals. First of all, about one-third of the STI patients were non-Italian natives, the majority of which were South Americans; besides, in AMAB individuals, having a STI was more frequently associated with being a non-Italian native (OR 4.58, 95% CI 2.14–9.84; *p* < 0.001) in line with previous studies [5,10,24]. Even though several complex sociological factors might contribute to this phenomenon, many authors have hypothesized that for trans people, belonging to an ethnic minority may further increase the risk of discrimination and social marginalization [16,22,24], which have a primary role as risk factors for potentially self-detrimental behaviors in people belonging to sexual minorities [5,26,27]. In line with that, our study showed that unemployment, as a possible result of discrimination, was more frequent in trans people with STIs, according to data from several studies [7,16]. Economic instability and troubles in finding a job can turn to prostitution as a way to earn a living [22,24,27]. Indeed, as previously described [5,22,27], the present study showed that trans people reporting at least one STI had a history of sex work more frequently than trans people without STI, and sex work represented a risk factor for STI in the AMAB, but not in AFAB individuals. However, the lack of financial stability may not be the only risk factor for sex work: some AMAB trans people may seek the affirmation of their female gender identity through sexual intimacy with partners, primarily through unprotected sexual intercourse [22,24,28], exposing themselves to the risk of STIs. Additionally, the study participants with STIs reported more frequently a history of substance abuse (in particular, of injectable substances, such as opiates and cocaine), which might be a way to cope with several past or present psychosocial stressors and sex work [16,24]. Indeed, the injection of illicit drugs is per se a possible way of contracting some STIs, but it adds up to the risk when leading to a state of mental alteration during sex. More in general, according to our results, trans people with STI reported a significantly higher use of psychiatric medications, and depression was significantly more frequent in that subgroup. History of incarceration was significantly more common in the subgroup of trans people with STIs representing a risk factor for STIs in trans AMAB persons, with a possible correlation (which was not evaluated in the present study) with sex work, which is a major issue leading to problems with justice [16,24].

Finally, discrimination and transphobia have many other turn-ups on the risk of STIs in the transgender population. Indeed, AMAB trans people with at least one STI had more frequently a history of sexual abuse during adolescence. Victimization and forced sexual intercourse are frequently reported by AMAB trans people, according to the literature, as possible outcomes of discrimination, lack of social support, economic instability and transphobia [5,29], especially in younger individuals [30].

## 5. Limitations

Our study has several limitations. First, the data were collected through a face-to-face interview, so the information is mostly self-reported and possibly underestimated and the authors performed serological testing for STIs only in a small percentage of participants referring to the Centre of Florence. Performing a systematic serological screening of all patients referring to gender clinics would let the authors to describe the STI prevalence in a study sample that could be representative of the Italian trans population. Moreover, the study was conducted enrolling transgender people with GD and referring to gender clinics, while it is likely that a relevant part of the gender diverse population does not feel the need for professional assessment. Thus, our sample might not be fully representative of the transgender population. Secondly, when evaluating the possible risk factors for STI transmission, several important pieces of information were not available: for example, participants were not explicitly asked whether they had a history of injection substance use (including drugs, silicone and hormones), and specific questions about type and frequency of unprotected sex were not asked. That would have been particularly important to investigate the risk behaviors in AFAB trans individuals, in whom multiple sex partners and injection substance abuse might be primary risk factors for STI transmission [9,16,31]: that might be the topic of future research.

Furthermore, aiming at including a large sample of transgender people, our survey was implemented between 2007 and 2020. Social aspects, healthcare policies and prevention programs may have changed during this period, with possible variations in STIs prevalence trends across the years.

Finally, among evaluated STIs, syphilis serological screening was assessed though the use of VDRL and TPHA and syphilis diagnosis was made if at least one resulted positive. These serological tests are currently used in clinical practice, although limited by a significant rate of false positives.

## 6. Conclusions

The present multicenter study on a broad Italian transgender population describes a significant prevalence of STIs in the study sample, with AMAB transgender individuals suffering from STIs more frequently than AFAB ones. In the AMAB subgroup, several socio-demographic features, such as unemployment, commercial sex work, history of sexual abuse, history of substance abuse, justice problems and not being Italian native, were found to be associated with having a STI.

All the aforementioned issues might somehow be correlated with discrimination, stigma and social isolation experienced by trans people as a sexual minority, possibly limiting the access of trans individuals to competent medical care [22,24]. Thus, the present study, in line with the available literature, highlighted the need for social, medical and psychological support for this vulnerable population in order to promote sanitary education, appropriate screening and therapy for transgender people at risk of or affected by STI [9,15,32]. For example, transgender persons’ referral to gender clinics due to their request for GAHT should be an occasion for STI counselling and testing in those at risk, as previously suggested [9]. Moreover, sex education regarding STI prevention strategies should be provided in the same setting [9]. Besides, the public health system might provide easy access to STI testing and referral for the general population, since many gender variant individuals are not in need of professional transgender care [3].

The present study stressed that trans clients with a history of risk behaviors for STI should be screened for threatening infectious diseases in addition to HIV, possibly including also STI that were not considered in the present study, such as chlamydia and gonorrhea.

## Figures and Tables

**Table 1:** Sociodemographic characteristics of the sample.

	All (*n* = 705) % (*n*)	At Least One STI (*n* = 48) % (*n*)	No STI (*n* = 657) % (*n*)	*p*	HR [95% Confidence Interval]	Adjusted *p*
**Age**	30.81 ± 10.09	39.42 ± 8.42	30.19 ± 9.92	*p* < 0.0001		
**Assigned gender at birth**						
AMAB	53.5% (377)	87.5% (42)	51.0% (335)	*p* < 0.0001	5.436 [2.253–13.118]	*p* < 0.0001
AFAB	46.5% (328)	12.5% (6)	49.0% (322)			
**Gender affirming path**						
HT	39.1% (276)	83.0% (40)	35.9% (236)	*p* < 0.0001	0.164 [0.074–0.363]	*p* < 0.0001
GAS	11.0% (78)	25.0% (12)	10.0% (66)	*p* = 0.001	1.574 [0.743–3.336]	*p* = 0.236
**Non-italian natives**	11.2% (79)	31.3% (15)	9.7% (64)	*p* < 0.0001	4.465 [2.229–8.943]	*p* < 0.0001
**Marital status**						
Unmarried	91.6 (646)	77.1% (37)	92.7% (609)	*p* < 0.0001	0.515 [0.231–1.149]	*p* = 0.105
Married	3.1% (22)	7.0% (3)	2.9% (19)	*p*= 0.137	0.825 [0.207–3.291]	*p* = 0.785
Divorced	3.3% (23)	4.7% (2)	3.2% (21)	*p* = 0.606	0.741 [0.159–3.441]	*p* = 0.701
Widowed	0.3% (2)	2.3% (1)	0.2% (1)	*p* = 0.01	10.696 [0.530–215.9]	*p* = 0.122
**Parenthood**	5.4% (38)	2.3% (1)	5.6% (37)	*p* = 0.342	0.078 [0.010–0.638]	*p* = 0.017
**Education**						
Primary school	26.5% (187)	31.9% (15)	26.1% (172)	*p* = 0.384	1.272 [0.657–2.461]	*p* = 0.476
Secondary school/professional diploma	58.7% (414)	51.1% (25)	59.3% (389)	*p* = 0.268	0.860 [0.465–1.592]	*p* = 0.632
University	14.8% (104)	17.0% (8)	14.6% (96)	*p* = 0.650	0.913 [0.399–2.089]	*p* = 0.830
**Employment**						
Student	18.2% (128)	0.0% (0)	19.5% (128)	*p* = 0.001	0.000 [0.000-]	*p* = 0.996
Employed	58.1% (410)	66.0% (32)	57.5% (378)	*p* = 0.257	0.923 [0.479–1.781]	*p* = 0.811
Retired	1.7% (12)	4.3% (2)	1.5% (10)	*p* = 0.165	0.565 [0.103–3.095]	*p* = 0.511
Unemployed	23.9% (168)	34.0% (16)	23.2% (152)	*p* = 0.091	2.152 [1.109–4.175]	*p* = 0.023
**Living status**						
With family	45.9% (324)	12.8% (6)	48.3% (318)	*p* < 0.0001	0.268 [0.108–0.668]	*p* = 0.005
With partner	18.7% (132)	21.3% (10)	18.5% (122)	*p* = 0.636	0.788 [0.368–1.688]	*p* = 0.54
With flatmate	11.4% (80)	14.9% (7)	11.1% (73)	*p* = 0.428	1.864 [0.776–4.476]	*p* = 0.164
Alone	23.1% (163)	42.6% (20)	21.7% (143)	*p* = 0.001	1.552 [0.807–2.985]	*p* = 0.188
Institute	1.0% (7)	4.2% (2)	0.8% (5)	*p* = 0.03	5.292 [0.90–31.129]	*p* = 0.065
Prison	0.6% (4)	4.2% (2)	0.3% (2)	*p* = 0.001	9.614 [1.242–74.398]	*p* = 0.03
**Previous incarceration**	2.1% (15)	12.5% (6)	1.4% (9)	*p* < 0.0001	8.118 [2.352–28.022]	*p* = 0.001
**Sex work**	10.8% (76)	59.3% (28)	7.3% (48)	*p* < 0.0001	14.196 [6.719–29.991]	*p* < 0.0001
**Justice problems**	9.4% (66)	35.1% (17)	7.5% (49)	*p* < 0.0001	4.716 [2.168–10.258]	*p* < 0.0001

This table reports the main sociodemographic features of our sample as derived from patient history. Data are expressed as percentages, while the absolute number of subjects is reported in parentheses. Data have been adjusted for age. Logistic regression analysis after adjustment for age was performed. STI = sexually transmitted infection; HR = hazard ratio; AMAB = assigned male at birth; AFAB = assigned female at birth; HT = hormonal treatment; GAS = gender-affirming surgery.

**Table 2:** Differences in lifestyle and health status among transgender individual with and without self-reported STIs.

	All (*n* = 705) % (*n*)	At Least One STI (*n* = 48) % (*n*)	No STI (*n* = 657) % (*n*)	*p*	HR [95% Confidence Interval]	Adjusted *p*
**Smoking status**						
Ex-smoker	15.7% (111)	13.0% (6)	15.9% (104)	*p* = 0.610	0.664 [0.267–1.653]	*p* = 0.379
Current smoker	45.5% (321)	54.3% (26)	44.9% (295)	*p* = 0.215	1.435 [0.772–2.668]	*p* = 0.253
**Comorbilities**						
Cardiovascular disease	1.6% (11)	4.3% (2)	1.4% (9)	*p* = 0.128	1.525 [0.297–7.829]	*p* = 0.613
Diabetes	1.1% (8)	2.1% (1)	1.1% (7)	*p* = 0.512	1.389 [0.149–12.970]	*p* = 0.773
Dyslipidemia	1.4% (10)	2.1% (1)	1.2% (8)	*p* = 0.598	0.861 [0.101–7.322]	*p* = 0.891
Hypertension	2.1% (15)	4.3% (2)	2.0% (13)	*p* = 0.301	0.944 [0.195–4.572]	*p* = 0.943
Cerebrovascular disease	0.4% (3)	0.0% (0)	0.5% (3)	*p* = 0.641	0.000 [0.000-]	*p* = 0.999
**Current medication use**	2.7% (19)	4.3% (2)	2.6% (17)	*p* = 0.501	2.808 [0.597–13.212]	*p* = 0.191
Alcohol consumption *	1.8% (13)	2.2% (1)	1.9% (12)	*p* = 0.885	1.01 [0.122–8.343]	*p* = 0.993
**Lifetime substance abuse**	31.4% (221)	50.0% (24)	30.0% (197)	*p* = 0.003	2.552 [1.374–4.740]	*p* = 0.003
Alcohol	11.9% (84)	8.3% (4)	12.2% (80)	*p* = 0.492	0.788 [0.231–2.692]	*p* = 0.704
Cannabis	19.4% (137)	22.9% (11)	19.2% (126)	*p* = 0.668	1.504 [0.651–3.477]	*p* = 0.339
Opiates	3.1% (22)	18.7% (9)	2.0% (13)	*p* < 0.0001	8.673 [3.115–24.147]	*p* < 0.0001
Cocaine	8.8% (62)	27.1% (13)	7.5% (49)	*p* < 0.0001	4.608 [2.046–10.378]	*p* < 0.0001
Hallucinogens	2.1% (15)	2.1% (1)	2.1% (14)	*p* = 0.794	1.191 [0.148–9.573]	*p* = 0.870
Ketamine	1.1% (8)	0.0% (0)	1.2% (8)	*p* = 0.517	0.000 [0.000-]	*p* = 0.999
**Psychiatric disorders in the family**	27.7% (195)	25.5% (12)	27.8% (183)	*p* = 0.730	0.946 [0.470–1.903]	*p* = 0.876
**Sexual abuse**						
Childhood	6.8% (48)	6.4% (3)	6.9% (45)	*p* = 0.886	0.757 [0.220–2.603]	*p* = 0.659
Adolescence	4.7% (33)	14.9% (7)	4.0% (26)	*p* = 0.001	4.178 [1.642–10.626]	*p* = 0.003
**Self-harming behaviors**	28.0% (197)	29.2% (14)	27.8% (183)	*p* = 0.705	1.350 [0.687–2.652]	*p* = 0.384
**Bullying**						
Childhood	15.0% (106)	8.3% (4)	15.5% (102)	*p* = 0.215	0.693 [0.237–2.026]	*p* = 0.503
Adolescence	17.3% (122)	6.5% (3)	18.1% (119)	*p* = 0.046	0.519 [0.154–1.749]	*p* = 0.290
**Suicidal ideation**	51.9% (366)	58.3% (28)	51.4% (338)	*p* = 0.283	1.529 [0.819–2.857]	*p* = 0.183
**Current psychiatric medications**	11.3% (80)	25.0% (12)	10.3% (68)	*p* = 0.002	2.579 [1.237–5.380]	*p* = 0.012
Antidepressants	6.5% (46)	8.3% (4)	6.4% (42)	*p* = 0.555	1.108 [0.367–3.345]	*p* = 0.856
Antipsychotics	1.4% (10)	2.1% (1)	1.4% (9)	*p* = 0.680	1.249 [0.143–10.936]	*p* = 0.841
Mood stabilizers	2.6% (18)	4.2% (2)	2.4% (16)	*p* = 0.457	1.728 [0.349–8.556]	*p* = 0.503
Benzodiazepines	4.5% (32)	12.5% (6)	4.0% (26)	*p* = 0.006	3.072 [1.157–8.155]	*p* = 0.024
**Lifetime psychiatric disorders**	21.5% (152)	37.5% (18)	20.4% (134)	*p* = 0.010	1.988 [1.013–3.901]	*p* = 0.046
GAD	2.0% (14)	0.0% (0)	2.1% (14)	*p* = 0.339	0.000 [0.000-]	*p* = 0.999
Depression	16.0% (113)	33.3% (16)	14.8% (97)	*p* = 0.002	2.329 [1.154–4.701]	*p* = 0.018
Dysthymia	0.1% (1)	0.0% (0)	0.2% (1)	*p* = 0.790	0.000 [0.000-]	*p* = 1.000
Bipolar disorder	1.1% (8)	0.0% (0)	1.2% (8)	*p* = 0.479	0.000 [0.000-]	*p* = 0.999
Panic disorder	4.3% (30)	2.1% (1)	4.4% (29)	*p* = 0.510	0.368 [0.047–2.882]	*p* = 0.341
Sociophobia	0.4% (3)	0.0% (0)	0.5% (3)	*p* = 0.644	0.000 [0.000-]	*p* = 0.999
PTSD	0.3% (2)	0.0% (0)	0.3% (2)	*p* = 0.706	0.000 [0.000-]	*p* = 0.999
OCD	0.9% (6)	0.0% (0)	0.9% (6)	*p* = 0.513	0.000 [0.000-]	*p* = 0.999
Any personality disorder	0.6% (4)	4.2% (2)	0.3% (2)	*p* < 0.0001	13.176 [1.582–109.719]	*p* = 0.017
Anorexia nervosa	0.9% (6)	2.1% (1)	0.8% (5)	*p* = 0.319	4.585 [0.467–45.068]	*p* = 0.192

Data are expressed as percentages, while the absolute number of subjects is reported in parentheses. Data have been adjusted for age. Logistic regression analysis after adjustment for age was performed. * More than 2 alcohol units per day. STI = sexually transmitted infection; GAD = generalized anxiety disorder; PTSD = post-traumatic stress disorder; OCD = obsessive-compulsive disorder.

## Data Availability

Not applicable.
